# Effect of ultrasound-guided stellate ganglion block on intraoperative hemodynamics in laparoscopic partial adrenalectomy: a randomized clinical trial

**DOI:** 10.3389/fnins.2026.1966414

**Published:** 2026-10-26

**Authors:** Jingyi Nie, Huang Wang, Wenjing Zhang, Hui Wang, Pengqian Hao, Junchai Liu, Jiwen Shang, Weiwei Zhang

**Affiliations:** 1 Department of Anesthesiology, Shanxi Medical University, Taiyuan, China; 2 School of Basic Medical Sciences, Shanxi Medical University, Taiyuan, China; 3 Department of Anesthesiology, Third Hospital of Shanxi Medical University, Shanxi Bethune Hospital, Shanxi Academy of Medical Sciences, Tongji Shanxi Hospital, Taiyuan, China; 4 Department of Urology, Shanxi Bethune Hospital, Shanxi Academy of Medical Sciences, Tongji Shanxi Hospital, Third Hospital of Shanxi Medical University, Taiyuan, China

**Keywords:** adrenal tumors, general anesthesia, hemodynamic stability, laparoscopic adrenalectomy, randomized controlled trial, stellate ganglion block

## Abstract

**Purpose:**

This study aimed to evaluate the effect of ultrasound-guided stellate ganglion block (SGB) on intraoperative hemodynamic parameters in patients undergoing laparoscopic partial adrenalectomy.

**Patients and methods:**

A total of 72 patients with adrenal tumors scheduled for laparoscopic partial adrenalectomy were enrolled and randomly assigned in a 1:1 ratio to either the stellate ganglion block (SGB) group or the control group. The primary outcome was the incidence of intraoperative hypertension, defined as a systolic blood pressure (SBP) ≥180 mmHg or an increase of ≥30% in mean arterial pressure (MAP) from baseline. Secondary outcomes included MAP and heart rate (HR) measured at nine predefined perioperative time points, intraoperative vasoactive drug consumption, and the incidence of perioperative adverse events.

**Results:**

The incidence of intraoperative hypertension was significantly lower in the SGB group than in the control group (14.3% vs. 37.1%, *p* = 0.029). Compared with the control group, patients in the SGB group exhibited significantly lower MAP and HR at eight time points ranging from immediately after tracheal intubation until the end of surgery (*p* < 0.05). The intraoperative consumption of phentolamine was significantly lower in the SGB group than in the control group [0 (0–4) mg vs. 0 (0–0) mg; *p* = 0.008]. In contrast, the consumption of norepinephrine was significantly higher in the SGB group [0 (0–0) mg vs. 0 (0–8) mg; *p* = 0.004]. No statistically significant differences were observed between the two groups in the consumption of esmolol and atropine, or in the incidence of perioperative adverse events.

**Conclusion:**

Ultrasound-guided SGB lowers intraoperative hypertension incidence and phentolamine demand in laparoscopic partial adrenalectomy. Although the SGB group observed a slight increase in the intraoperative dose of norepinephrine, no perioperative adverse events occurred. There was also no significant difference in the incidence of adverse reactions between the two groups.

## 1 Introduction

Adrenal tumors are common neoplasms encountered in urological practice and predominantly include pheochromocytoma/paraganglioma (PPGL), primary aldosteronism (PA), Cushing’s syndrome (CS), and non-functioning adrenal adenomas (NFAs). Current epidemiological studies estimate the prevalence of adrenal tumors to be approximately 4–6% (Baston et al., 2025). Laparoscopic partial adrenalectomy features the merits of minimal invasiveness, mild postoperative pain, and expedited recovery, and has emerged as a widely adopted clinical procedure (Kwak and Lee, 2020). However, surgical manipulation of the tumor can provoke catecholamine release, which induces substantial hemodynamic fluctuations (Nizamoğlu et al., 2011). In addition, carbon dioxide pneumoperitoneum increases both systemic vascular resistance and arterial blood pressure, exacerbating these hemodynamic perturbations (Lenz et al., 1976). As such, maintenance of intraoperative hemodynamic stability constitutes a key challenge in the anesthetic management of these patients.

The stellate ganglion (SG) is a sympathetic structure formed by the fusion of the inferior cervical and first thoracic ganglia. It is typically located anterior to the transverse processes of the C6 and C7 vertebrae and is a key component of the cervical sympathetic nervous system (Duan et al., 2024). Stellate ganglion block (SGB) is performed by injecting local anesthetic into the tissue surrounding the SG. Currently, SGB is widely used in the clinical management of various conditions, including perioperative arrhythmias (Nunez-Rodriguez et al., 2026), perimenopausal hot flashes (Li et al., 2023), sleep disorders (Zhang et al., 2024), and post-traumatic stress disorder (PTSD) (Stein, 2020). Previous studies have demonstrated that SGB can modulate the activity of the hypothalamic–pituitary–adrenal (HPA) axis, thereby regulating adrenal hormone secretion (Wang et al., 2017).

Based on this pathophysiological and pharmacological rationale, we hypothesized that preoperative ultrasound-guided SGB would maintain intraoperative hemodynamic stability in patients with adrenal tumors undergoing laparoscopic partial adrenalectomy by attenuating sympathetic activity and modulating adrenal hormone secretion. Accordingly, we conducted a randomized controlled trial to evaluate the effect of preoperative ultrasound-guided SGB on intraoperative hemodynamics in this patient population.

## 2 Materials and methods

### 2.1 Trial design

This single-center, prospective, randomized controlled trial was conducted at Shanxi Bethune Hospital. The study protocol was approved by the Clinical Research Ethics Committee of Shanxi Bethune Hospital (Approval No. YXLL-2025-033) and prospectively registered in the Chinese Clinical Trial Registry (Registration No. ChiCTR2500113024; http://www.chictr.org.cn). The trial was conducted in accordance with the Consolidated Standards of Reporting Trials (CONSORT) guidelines. Written informed consent was obtained from all participants prior to enrollment.

### 2.2 Patients

Inclusion criteria were as follows: (1) male or female patients aged 18–65 years without fertility requirements; (2) a preoperative diagnosis of aldosteronism, hypercortisolism, or non-functioning adrenal adenoma confirmed by laboratory tests and imaging examinations; (3) meeting the surgical indications for laparoscopic partial adrenalectomy (only the tumor lesion is resected, with the remaining functional adrenal cortex preserved, instead of performing a unilateral total adrenalectomy); and (4) voluntary participation in the study with provision of written informed consent.

Exclusion criteria were as follows: (1) New York Heart Association (NYHA) functional class III or IV; (2) a resting heart rate <50 beats per minute (bpm); (3) an anticipated need for postoperative treatment in an intensive care unit (ICU); (4) an anticipated difficult airway; (5) pregnancy or lactation; and (6) participation in another interventional clinical trial within the 3 months preceding enrollment.

Several studies have demonstrated that SGB attenuates the elevation of blood catecholamine levels induced by carbon-dioxide pneumoperitoneum (Chen et al., 2017). In contrast, pheochromocytoma resection itself may cause a precipitous decline in circulating catecholamines, which predisposes patients to severe postoperative hypotension (Namekawa et al., 2016). Given these safety considerations, patients diagnosed with pheochromocytoma were excluded from the present study.

### 2.3 Randomization and blinding

A computer-generated randomization sequence was created using SPSS software (version 25.0; IBM Corp., Armonk, NY, USA), and eligible patients were randomly assigned in a 1:1 ratio to either the SGB group or the control group. Group allocation was performed by research staff who were not involved in intraoperative anesthetic management or postoperative statistical analysis. This study employed a single-blind design. The anesthesiologist performing the SGB was the only individual aware of group assignments. The anesthesiologists responsible for intraoperative management, operating room nursing staff, surgeons, and patients were all blinded to group allocation.

In the SGB group, ultrasound-guided SGB was performed 20 min prior to induction of general anesthesia in a laminar-flow operating room under strict aseptic conditions (Jiang et al., 2025). The patient was placed in a supine position without a pillow, with the head rotated to the contralateral side. A linear ultrasound probe (10 MHz; LOGIQ 5, GE Healthcare) was used to scan the affected side. At the level of the cricoid cartilage, the internal carotid artery and internal jugular vein were identified. The SG was identified on the surface of the longus colli muscle, deep to the internal carotid artery. The puncture site was localized anterior to the transverse process of the sixth cervical (C6) vertebra. Following negative aspiration to confirm the absence of cerebrospinal fluid, blood, or air, 3 mL of 1% lidocaine was slowly injected to a target depth of 3–3.5 cm. The development of Horner’s syndrome (manifested as miosis, ptosis, enophthalmos, and conjunctival hyperemia) on the ipsilateral side was considered indicative of a successful block.

Patients in the control group underwent a blinded sham procedure. Under ultrasound guidance, the stellate ganglion region was localized, and a puncture needle was inserted without fluid injection, thereby simulating the SGB procedure. The anesthesiologist performing the SGB or sham procedure was responsible solely for the ultrasound-guided puncture and was not involved in subsequent intraoperative management or other trial procedures.

### 2.4 Anesthesia management

Peripheral venous access was established in all patients prior to entering the operating room. Upon arrival in the operating room, standard monitoring was initiated, including continuous electrocardiography, pulse oximetry (SpO_2_), end-tidal carbon dioxide (EtCO_2_), and bispectral index (BIS) monitoring. Given the high risk of intraoperative hemodynamic fluctuations, invasive arterial blood pressure monitoring was established in all patients prior to induction of anesthesia to enable real-time blood pressure monitoring.

To prevent postoperative nausea and vomiting, 0.12 mg of palonosetron and 5 mg of dexamethasone were administered intravenously 3 min prior to induction of anesthesia. Subsequently, all patients underwent preoxygenation with 100% oxygen at a flow rate of 3 L/min. Anesthesia was induced with intravenous etomidate, sufentanil (0.4 μg/kg), and rocuronium (0.8 mg/kg). Following loss of consciousness, mask ventilation with continuous positive pressure was initiated. The oxygen flow rate was increased to 4 L/min, and ventilation was set to a tidal volume of 6 mL/kg based on ideal body weight. Two minutes after rocuronium administration, tracheal intubation was performed under video laryngoscopic guidance. The patient was then connected to the anesthesia machine for volume-controlled mechanical ventilation.

The inspired oxygen concentration was maintained at 50%. Anesthesia was maintained with continuous intravenous infusions of propofol (0.2–0.6 mg/kg/h) and remifentanil (3–15 μg/kg/h), combined with inhaled sevoflurane at an end-tidal concentration of 0.8 minimum alveolar concentration (MAC). The depth of anesthesia was titrated to maintain the bispectral index (BIS) between 40 and 60. Infusion rates were adjusted as follows: if the BIS exceeded 60, the propofol infusion rate was increased by 1 mg/kg/h and the remifentanil infusion rate by 0.01 μg/kg/min; if the BIS was below 40, the infusion rates of both agents were decreased proportionally. Muscle relaxation was maintained with intermittent intravenous boluses of rocuronium (10 mg every 30 min), with dose and timing adjusted as needed based on surgical conditions. Sevoflurane administration was discontinued upon removal of the adrenal tumor specimen. Propofol and remifentanil infusions were terminated upon closure of the surgical incision. Following completion of the surgical procedure, sugammadex sodium (2 mg/kg) was administered intravenously to reverse residual neuromuscular blockade induced by rocuronium.

One minute after establishment of invasive arterial blood pressure monitoring in the operating room, the recorded mean arterial pressure (MAP) and heart rate (HR) were defined as baseline values. All surgical procedures were performed with patients in the lateral decubitus position. Hemodynamic abnormalities were defined and managed according to the following protocol. Hypotension, defined as a systolic blood pressure (SBP) <80 mmHg or a ≥30% decrease in MAP from baseline, was treated with an immediate intravenous bolus of norepinephrine (4 μg), repeated as needed. Hypertension, defined as an SBP ≥ 180 mmHg or a ≥30% increase in MAP from baseline, was treated with an immediate intravenous bolus of phentolamine (2 mg), repeated as needed. Bradycardia, defined as an HR < 50 bpm, was treated with an intravenous bolus of atropine (0.25 mg). Tachycardia, defined as an HR > 100 bpm, was treated with an intravenous bolus of esmolol (10 mg) as clinically indicated. Administration of all specified vasoactive and antiarrhythmic agents could be repeated intraoperatively as clinically required.

### 2.5 Outcome variables

The primary outcome was the incidence of intraoperative hypertension, defined as the occurrence of an SBP ≥ 180 mmHg or a ≥30% increase in MAP from baseline at any time during surgery. Secondary outcomes included intraoperative hemodynamic parameters, vasoactive drug consumption, and the incidence of perioperative hemodynamic abnormalities and adverse events. Hemodynamic parameters included mean arterial pressure (MAP) and heart rate (HR) recorded at nine predefined time points: T0 (1 min after establishment of invasive arterial monitoring, baseline), T1 (immediately after tracheal intubation), T2 (start of surgery), T3 (after establishment of pneumoperitoneum), T4 (10 min after initiation of tumor exploration), T5 (30 min after surgery onset), T6 (50 min after surgery onset), T7 (tumor specimen removal), and T8 (end of surgery). Drug consumption was defined as the total intraoperative dose of phentolamine, esmolol, norepinephrine, and atropine administered.

### 2.6 Statistical analysis

The normality of continuous variables was assessed using the Shapiro–Wilk test. Normally distributed continuous variables are presented as mean (standard deviation [SD]) and were compared between groups using the independent-samples Student’s *t*-test. Non-normally distributed continuous variables are presented as median (interquartile range [IQR]) and were compared between groups using the Mann–Whitney *U* test. Categorical variables are presented as number (percentage) and were analyzed using the chi-square test or Fisher’s exact test, as appropriate.

For repeated-measures data, such as MAP and HR recorded at multiple intraoperative time points, a linear mixed-effects model was used to analyze differences in hemodynamic trends over time between the SGB and control groups. The model included a random intercept for each subject, with time, treatment group, and the time-by-group interaction as fixed effects. A two-tailed *p* value < 0.05 was considered statistically significant. All statistical analyses were performed using SPSS version 25.0.

### 2.7 Sample size

Based on the results of a pilot study (15 patients per group), the sample size was calculated using the incidence of intraoperative hypertension as the primary outcome. In the pilot study, the incidence of intraoperative hypertension was 33.3% in the control group and 6.7% in the SGB group. With a two-sided significance level (*α*) of 0.05, a power (1 − *β*) of 0.8, and an anticipated dropout rate of 10%, the required sample size was calculated as 36 participants per group, yielding a total of 72 participants. The sample size calculation was performed using PASS version 15 (NCSS, LLC, Kaysville, UT, USA).

## 3 Results

A total of 80 patients with adrenal tumors were assessed for eligibility. Eight patients were excluded: three had pheochromocytoma, two had an anticipated difficult airway, and three declined participation. Consequently, 72 patients were enrolled and randomly assigned. Following randomization, one patient in the control group was excluded due to conversion to open surgery, and one patient in the SGB group was excluded after declining treatment upon arrival in the operating room. The remaining 70 patients (35 in the SGB group and 35 in the control group) completed the study protocol (Figure 1).

**Figure 1:**
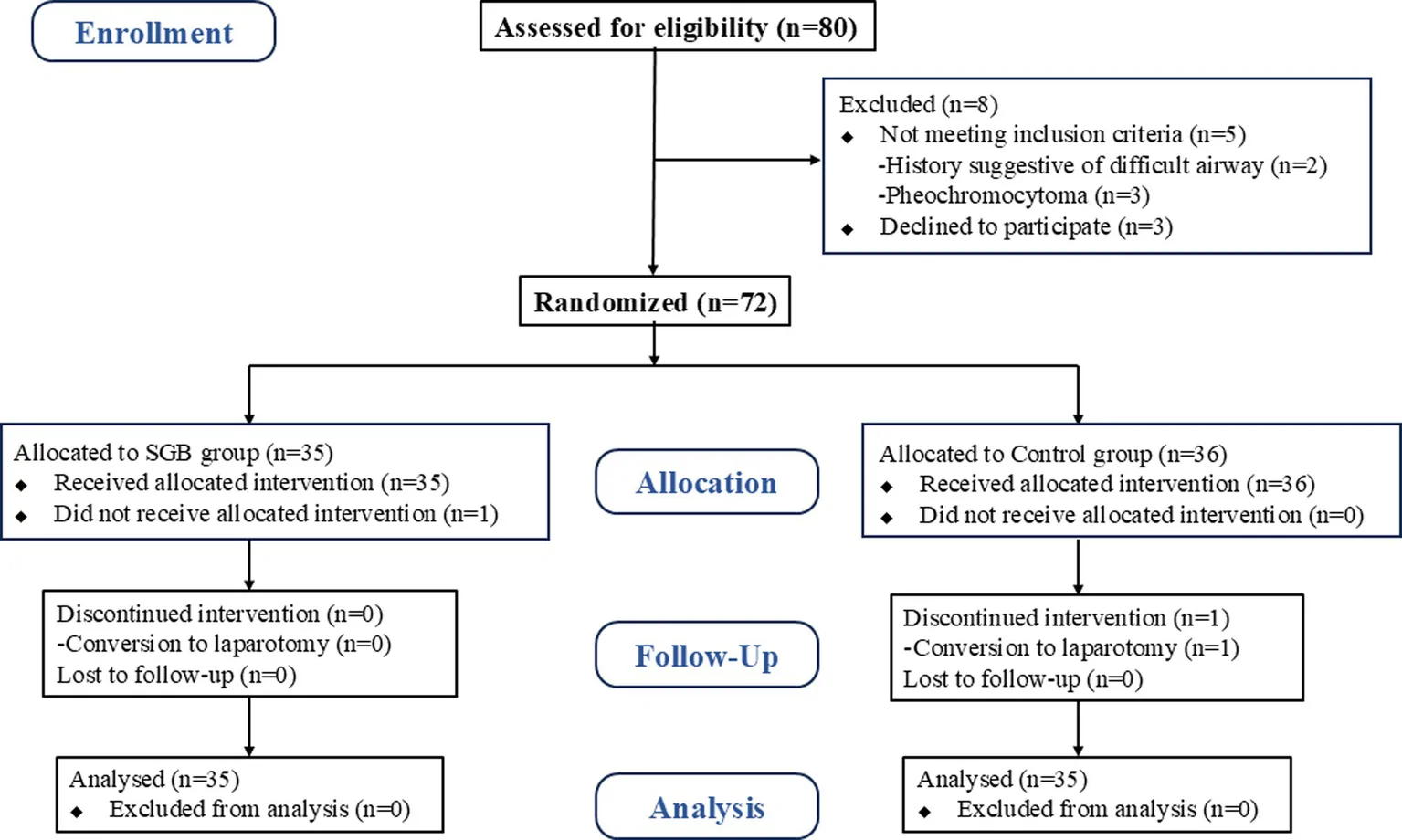
CONSORT flow chart of the trial.

Comparison of baseline characteristics revealed no statistically significant differences between the SGB and control groups in terms of age, sex, height, weight, body mass index (BMI), duration of surgery, baseline mean arterial pressure (MAP), or baseline heart rate (HR) (all *p* > 0.05), confirming baseline comparability between the two groups (Table 1).

**Table 1:** Patient characteristics.

	Control group (*n* = 35)	SGB group (*n* = 35)	*p* value
Age (years)	45.91 (10.77)	48.57 (10.23)	0.294
Height (m)	1.68 (0.067)	1.68 (0.065)	0.843
Weight (kg)	68.92 (9.30)	67.58 (8.38)	0.530
BMI (kg/m^2^)	24.46 (4.60)	23.92 (1.83)	0.254
Female	16 (45.7%)	13 (37.1%)	0.499
ASA classification			
II	33 (94.3%)	29 (82.9%)	0.133
III	2 (5.7%)	6 (17.1%)	
Smoking history	15 (42.0%)	14 (40%)	0.808
Hypertension	30 (85.7%)	28 (80.0%)	0.526
Diabetes	9 (25.7%)	4 (11.4%)	0.124
Coronary heart disease	3 (8.6%)	5 (14.3%)	0.452
Patient type			
Hyperaldosteronism	21 (60.0%)	23 (62.9%)	0.805
Cushing’s syndrome	3 (8.6%)	2 (5.7%)	>0.999
Non-functional adenoma	11 (31.4%)	10 (28.6%)	>0.999
Tumor diameter (cm)	1.96 (0.91)	1.68 (0.91)	0.216
Surgery time (min)	98.6 (14.41)	94.17 (14.75)	0.208
baseline HR (bpm)	82.29 (6.22)	80.31 (6.68)	0.206
baseline MAP (mmHg)	93.34 (8.94)	97.60 (10.11)	0.066

Data are shown as mean (SD), interquartile ranges (IQR), or *n* (%). ASA, American Society of Anesthesiologists; BMI, body mass index, SGB, Stellate Ganglion Block.

### 3.1 Comparison of MAP at different time points between the two groups

Compared with baseline (T0), MAP in the control group increased significantly immediately after tracheal intubation (T1) (*p* < 0.001) and decreased significantly at tumor removal (T7) and at the end of surgery (T8) (*p* = 0.025 and *p* = 0.001, respectively), with no statistically significant differences at other intraoperative time points (all *p* > 0.05). In the SGB group, MAP was significantly lower than baseline at T2 (start of surgery), T3 (after establishment of pneumoperitoneum), T4 (10 min after initiation of tumor exploration), T5 (30 min after surgery onset), T6 (50 min after surgery onset), T7 (tumor specimen removal), and T8 (end of surgery) (all *p* < 0.001) (Table 2).

**Table 2:** Mean arterial blood pressure changes from the baseline in SGB and control groups.

Time from baseline	MD	SE	Lower	Upper	*p*	MD	SE	Lower	Upper	*p*
	Control group					SGB group				
T1	10.91	1.84	14.53	7.30	<0.001	−0.80	1.93	2.99	−4.59	0.678
T2	2.46	1.84	6.07	−1.16	0.182	−11.09	1.93	−7.30	−14.87	<0.001
T3	−0.97	1.84	2.65	−4.59	0.600	−12.49	1.93	−8.70	−16.27	<0.001
T4	−1.66	1.84	1.96	−5.27	0.368	−13.66	1.93	−9.87	−17.44	<0.001
T5	−0.71	1.84	2.90	−4.33	0.698	−12.66	1.93	−8.87	−16.44	<0.001
T6	−0.37	1.84	3.25	−4.00	0.840	−13.37	1.93	−9.59	−17.16	<0.001
T7	−6.11	1.84	−0.53	−7.76	0.025	−14.00	1.93	−10.21	−17.79	<0.001
T8	−8.77	1.84	−2.50	−9.73	0.001	−16.03	1.93	−12.24	−19.82	<0.001

MD, mean difference; SE, standard error; SGB, Stellate Ganglion Block.

Intergroup comparisons revealed that MAP in the SGB group was significantly lower than in the control group at all measured time points (T1–T8), with all differences reaching statistical significance (*p* = 0.009, *p* < 0.001, *p* = 0.004, *p* = 0.004, *p* = 0.015, *p* = 0.002, *p* = 0.001, and *p* = 0.006) (Figure 2).

**Figure 2:**
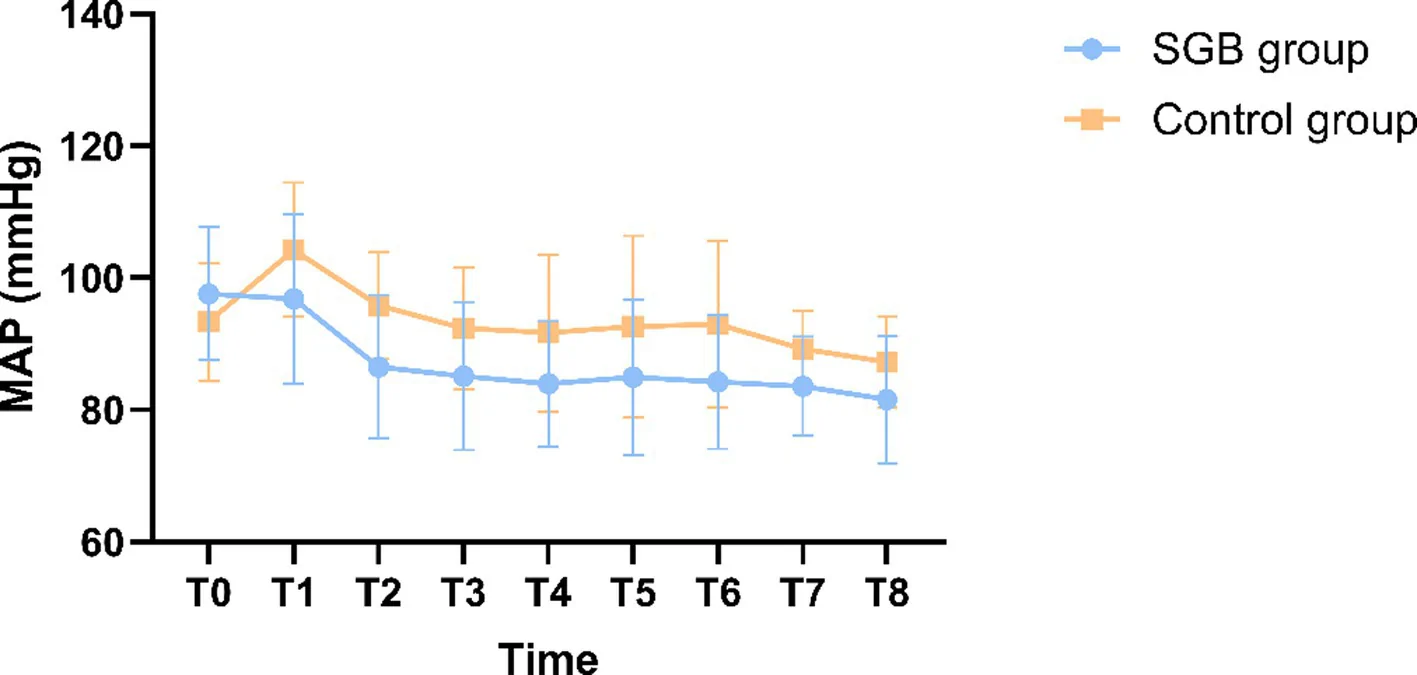
Perioperative median (IQR) mean arterial pressure. MAP, mean arterial pressure; SGB, Stellate Ganglion Block.

### 3.2 Comparison of HR at different time points between the two groups

Compared with baseline (T0), heart rate (HR) in the control group increased significantly immediately after tracheal intubation (T1) (*p* < 0.001) but did not differ significantly at the start of surgery (T2) (*p* = 0.150). HR was significantly lower than baseline at T3 (after establishment of pneumoperitoneum; *p* = 0.003), T5 (30 min after surgery onset; *p* < 0.001), T6 (50 min after surgery onset; *p* < 0.001), T7 (tumor specimen removal; *p* < 0.001), and T8 (end of surgery; *p* < 0.001). In the SGB group, HR at T1 (immediately after intubation) did not differ significantly from baseline (*p* = 0.317). However, HR was significantly lower than baseline at T3, T5, T6, T7, and T8 (all *p* < 0.001) (Table 3).

**Table 3:** Heart rate changes from the baseline in SGB and control groups.

Time from induction	MD	SE	Lower	Upper	*p*	MD	SE	Lower	Upper	*p*
	Control group					SGB group				
T1	7.17	0.79	8.73	5.61	<0.001	0.86	0.86	2.54	−0.83	0.317
T2	1.14	0.79	2.70	−0.41	0.150	−5.37	0.86	−3.69	−7.06	<0.001
T3	−2.57	0.79	−1.01	−4.13	0.001	−8.71	0.86	−7.03	−10.40	<0.001
T4	−5.14	0.79	−3.59	−6.70	<0.001	−10.34	0.86	−8.66	−12.03	<0.001
T5	−6.51	0.79	−4.96	−8.07	<0.001	−10.43	0.86	−8.74	−12.11	<0.001
T6	−7.29	0.79	−5.73	−8.84	<0.001	−10.74	0.86	−9.06	−12.43	<0.001
T7	−7.49	0.79	−5.93	−9.04	<0.001	−9.54	0.86	−7.86	−11.23	<0.001
T8	−6.97	0.79	−5.41	−8.53	<0.001	−9.17	0.86	−7.49	−10.86	<0.001

MD, mean difference; SE, standard error; SGB, Stellate Ganglion Block.

Intergroup comparisons revealed that heart rate (HR) in the SGB group was significantly lower than in the control group at all intraoperative time points (T1–T8): T1 (*p* < 0.001), T2 (*p* < 0.001), T3 (*p* < 0.001), T4 (*p* < 0.001), T5 (*p* < 0.001), T6 (*p* < 0.001), T7 (*p* = 0.004), and T8 (*p* = 0.001) (Figure 3).

**Figure 3:**
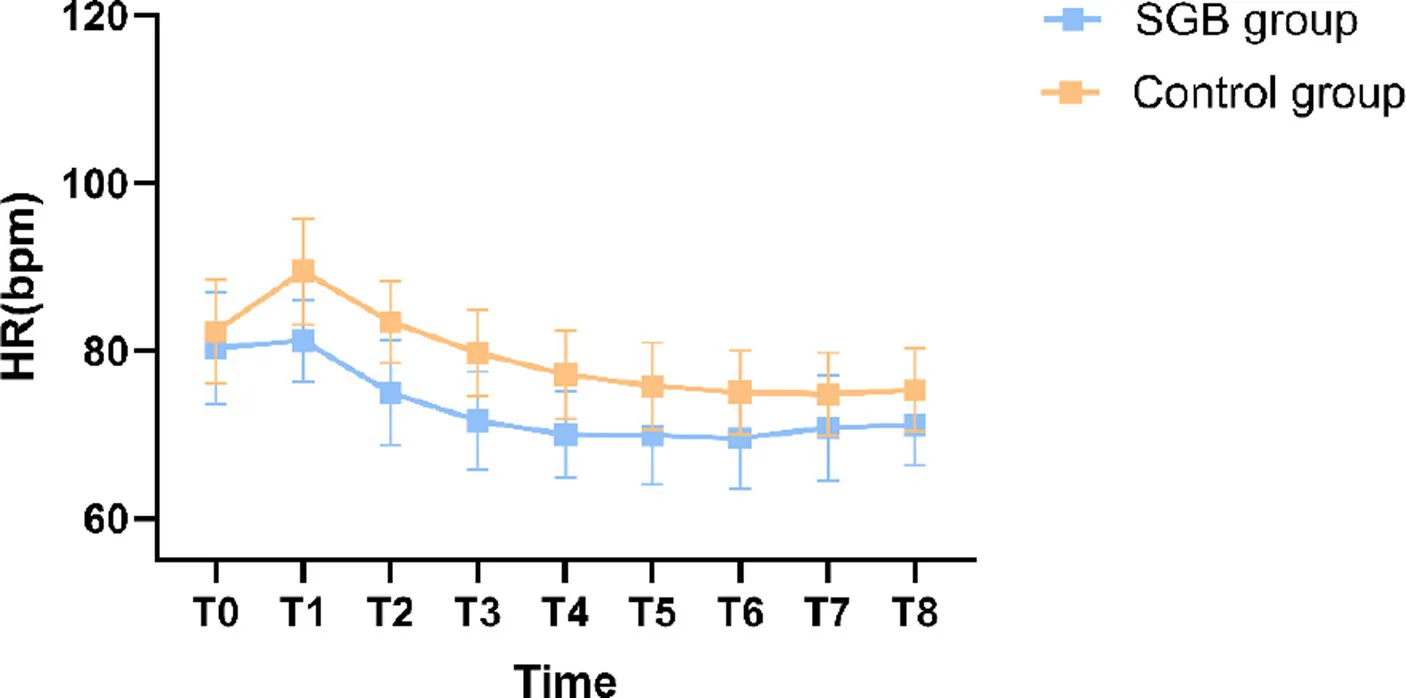
Perioperative median (IQR) heart rate. HR, heart rate; SGB, Stellate Ganglion Block.

### 3.3 Incidence of intraoperative hypertension, use of vasoactive drugs and incidence of adverse reactions between the two groups

The incidence of intraoperative hypertension was significantly lower in the SGB group (14.3%) than in the control group (37.1%) (*p* = 0.029) (Table 4).

**Table 4:** Incidence of hemodynamic instability events and dosages of vasoactive drugs.

	Control group (*n* = 35)	SGB group (*n* = 35)	*p* value
Intraoperative hypertension, No.	13 (37.1%)	5 (14.3%)	0.029
Phentolamine dose (mg)	0.0 (0–4.0)	0.0 (0.0–0.0)	0.008
Hypotension, No.	2 (5.7%)	12 (34.3%)	0.006
Norepinephrine dose (μg)	0 (0.0–0.0)	0.0 (0.0–8.0)	0.004
Tachycardia, No.	5 (14.3%)	2 (5.7%)	0.428
Esmolol dose (mg)	0.0 (0.0–0.0)	0.0 (0.0–0.0)	0.397
Bradycardia, No.	5 (14.3%)	6 (17.1%)	*p* > 0.999
Atropine (mg)	0.0 (0.0–0.0)	0.0 (0.0–0.0)	0.745

Intraoperative phentolamine consumption was significantly lower in the SGB group than in the control group (*p* = 0.008). In contrast, norepinephrine consumption was significantly higher in the SGB group (*p* = 0.004). No statistically significant differences were observed between the groups in intraoperative esmolol or atropine consumption (both *p* > 0.05) (Figure 4). Similarly, the incidence of intraoperative adverse events did not differ significantly between the groups (*p* > 0.05) (Table 5).

**Figure 4:**
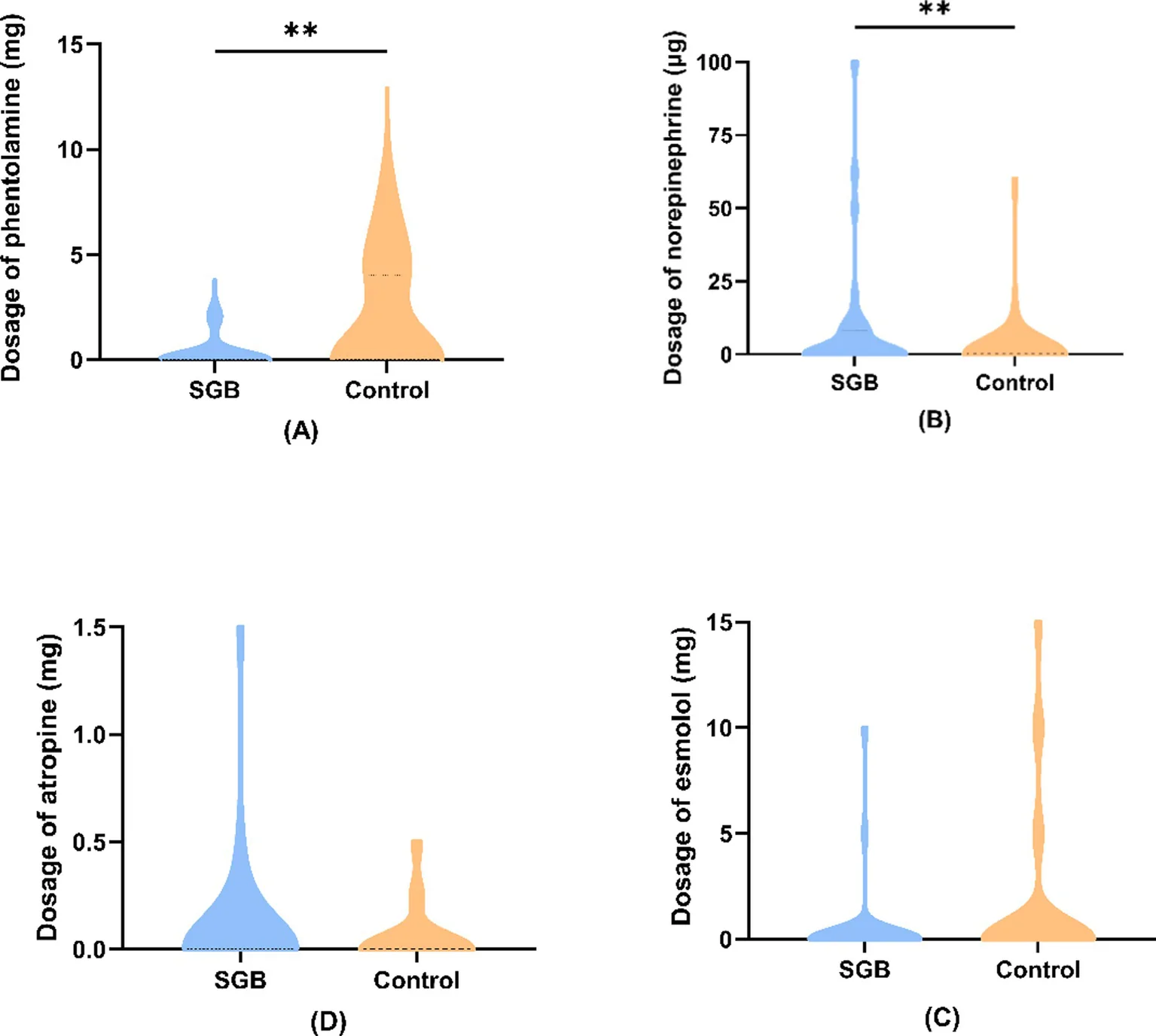
The usage of perioperative vasoactive drugs. **(A)** Dosage of phentolamine; **(B)** Dosage of norepinephrine; **(C)** Dosage of esmolol; **(D)** Dosage of atropine.

**Table 5:** Incidence of postoperative adverse reactions.

	Control group (*n* = 35)	SGB group (*n* = 35)	*p* value
Nausea	2 (5.71%)	1 (2.86%)	0.346
Hoarseness	0 (0%)	1 (2.86%)	0.057
Sore throat	1 (5.71%)	1 (5.71%)	*p* > 0.999

All patients in the SGB group developed transient ipsilateral Horner’s syndrome after SGB, and no case of persistent postoperative Horner’s syndrome was observed. Only one patient in the SGB group presented transient hoarseness, which fully resolved within 2 h after surgery.

## 4 Discussion

In this single-center randomized controlled trial, we investigated the effects of preoperative ultrasound-guided SGB on intraoperative hemodynamics in patients undergoing laparoscopic partial adrenalectomy. The results demonstrated that, compared with standard anesthetic management, preoperative SGB significantly reduced mean arterial pressure (MAP) and heart rate (HR) at all measured intraoperative time points, lowered the incidence of intraoperative hypertension, reduced the intraoperative requirement for the antihypertensive agent phentolamine, and did not increase the risk of intraoperative adverse events. These findings indicate that SGB exerts a regulatory effect on perioperative hemodynamics, offering a potential clinical strategy for anesthetic management in this type of surgery.

Severe intraoperative hemodynamic fluctuations during laparoscopic partial adrenalectomy primarily result from a dual stress response induced by the combined effects of the tumor’s inherent pathophysiology and the surgical intervention. First, adrenal tumors can secrete abnormal levels of hormones, such as aldosterone and cortisol. Intraoperative manipulation, including traction or compression of the tumor, can trigger the abrupt and massive release of these hormones, resulting in intense vasoconstriction and a precipitous increase in blood pressure (Nizamoğlu et al., 2011). Second, the creation of a CO_2_ pneumoperitoneum during laparoscopic surgery introduces additional physiological stress. Absorbed CO_2_ contributes to systemic hypercapnia and acidosis, while elevated intra-abdominal pressure compresses the abdominal aorta and impairs venous return (Lin et al., 2025). This hemodynamic disturbance further enhances sympathetic activity, promoting the release of catecholamines and other stress mediators. This cascade exacerbates fluctuations in blood pressure and heart rate, thereby increasing the risk of severe perioperative complications, including myocardial ischemia and cerebrovascular events.

SGB may improve intraoperative hemodynamics by inhibiting sympathetic activity and modulating the systemic stress response through several mechanisms. First, as a key junction in the cervicothoracic sympathetic chain, SGB can directly inhibit sympathetic efferent impulses, thereby reducing the release of stress hormones such as norepinephrine (Liu et al., 2025). This leads to decreased vascular smooth muscle activity, attenuation of vasoconstriction, and a consequent reduction in peripheral vascular resistance. Second, SGB may modulate the activity of the hypothalamic–pituitary–adrenal (HPA) axis, thereby inhibiting excessive adrenal hormone secretion and reducing hemodynamic fluctuations at their source (Wang et al., 2017). Third, by providing a preemptive blockade of sympathetic conduction, SGB can inhibit reflex sympathetic activation triggered by intraoperative tumor manipulation (Yuan et al., 2023), thereby attenuating severe fluctuations in blood pressure and heart rate and potentially reducing the risk of target organ damage.

Our findings are consistent with those of previous clinical studies. For instance, Xue et al. demonstrated that SGB reduces intraoperative MAP and HR in elderly patients undergoing gastrointestinal surgery, thereby maintaining hemodynamic stability (Yan et al., 2023). Similarly, Chen et al. reported that SGB attenuates the hemodynamic response to tracheal intubation and reduces fluctuations induced by surgical stress in elderly patients (Chen et al., 2015). Another study by Lao et al. further demonstrated that SGB reduces MAP fluctuations in patients undergoing partial hepatectomy (Lao et al., 2024). In contrast, other studies have reported that SGB has no significant effect on perioperative hemodynamics in patients undergoing laparoscopic gynecological surgery (Rahimzadeh et al., 2020). This discrepancy may be primarily attributed to differences in the pathophysiological status of the study populations. Intraoperative traction of the adrenal gland may elicit a stress response, promoting robust catecholamine release and subsequent marked hemodynamic lability (Nizamoğlu et al., 2011). SGB can inhibit this heightened sympathetic tone and reduce the release of stress mediators, thereby exerting a pronounced hemodynamic modulatory effect. Additionally, the anatomical proximity of the stellate ganglion and vagus nerve fibers within a shared connective tissue sheath may result in mild vagal blockade during SGB (Bookout and Gautron, 2021; Mathkour et al., 2022; Kronsteiner et al., 2024). However, compared with its dominant sympathetic inhibitory effect, the vagal influence is likely minimal (Puente de la Vega Costa et al., 2016). This predominant sympathetic inhibition provides a plausible explanation for the significant reductions in MAP and HR observed in the SGB group in our study.

A noteworthy finding of this study is the significantly greater intraoperative consumption of norepinephrine in the SGB group than in the control group. The potential mechanisms underlying this observation may involve two aspects. First, by inhibiting sympathetic efferent impulses, SGB induces relaxation of peripheral vascular smooth muscle and reduces peripheral vascular resistance. This physiological effect lowers blood pressure, thereby increasing the requirement for intraoperative vasopressor support. Second, although SGB has been shown to alleviate visceral pain during laparoscopic surgery (Lin et al., 2025; Xiang et al., 2024), pain perception was not specifically monitored in this study. Consequently, the depth of analgesia may have been overestimated, potentially resulting in a relatively high remifentanil dose, exacerbating intraoperative hypotension and contributing to increased norepinephrine use. Despite the increased norepinephrine requirement in the SGB group, the incidence of intraoperative adverse events did not differ significantly from that in the control group. These findings indicate that, with standardized anesthetic management and appropriate vasoactive drug titration, the application of SGB during the perioperative period of laparoscopic partial adrenalectomy is safe.

This study has several limitations that should be addressed in future research. First, as a single-center study with a limited sample size, the findings may be subject to selection bias. The generalizability and external validity of these findings require confirmation in future multicenter, large-scale randomized controlled trials. Second, patients with pheochromocytoma, who are at high risk of post-resection hypotension, were excluded for safety reasons because SGB inhibits the stress response. Consequently, the effects of SGB in this specific population remain to be investigated. Third, this study did not perform pre-specified stratified subgroup analysis according to adrenal tumor subtypes (e.g., aldosterone-producing adenoma, cortisol-producing adenoma and non-functioning adenoma). Differences in hormone-secreting patterns and baseline sympathetic tone among tumor subtypes might lead to variable responses to SGB. Fourth, this study focused solely on intraoperative hemodynamics. The lack of follow-up data on postoperative cardiovascular function and quality of life precludes assessment of the long-term benefits of SGB.

In light of these limitations, future research may be directed along several avenues. First, multicenter, large-scale prospective randomized controlled trials recruiting participants from diverse geographical regions and healthcare settings are warranted to further validate the perioperative efficacy of SGB in laparoscopic adrenal surgery. Second, stratified analyses based on the pathological subtypes of adrenal tumors are needed to clarify the differential efficacy of SGB across patient subgroups, thereby informing the development of individualized block regimens. Third, future adequately powered multicenter randomized trials with pre-defined subgroup hypotheses are required to clarify potential subtype-specific effects of SGB. Fourth, extending the follow-up period would allow evaluation of the effects of SGB on both short- and long-term postoperative cardiovascular function and patient quality of life. Finally, under rigorous anesthetic protocols and safety monitoring, inclusion of patients with pheochromocytoma would enable investigation of the hemodynamic regulatory effects and safety profile of SGB in this high-risk population.

## 5 Conclusion

Preoperative ultrasound-guided SGB effectively reduces the risk of intraoperative hypertension in patients undergoing laparoscopic partial adrenalectomy. It offers a potential strategy for optimizing perioperative anesthetic management for this patient population without elevating the risk of intraoperative adverse events.

## Data Availability

The raw data supporting the conclusions of this article will be made available by the authors, without undue reservation.
